# The Influence of Incretin Mimetics on Cardiovascular Risk Factors in Diabetes

**DOI:** 10.5402/2012/625809

**Published:** 2012-02-15

**Authors:** Ida Kinalska, Dorota Bednarska-Chabowska, Joanna Adamiec-Mroczek, Łukasz Hak

**Affiliations:** ^1^Clinic of Endocrinology Diabetology and Internal Medicine, Medical University of Bialystok, M.Curie-Sklodowska 24a, 15-269 Bialystok, Poland; ^2^Clinic of Angiology, Arterial Hypertension and Diebetology, Medical Academy in Wroclaw, ul. Poniatowskiego 2, 50-326 Wrocław, Poland; ^3^Clinic of Ophthalmology, Medical Academy in Wroclaw, ul. Borowska 213, 50-556 Wrocław, Poland; ^4^Novo Nordisk Pharma Sp. z o.o., ul. 17 Stycznia 45B, 02-146 Warszawa, Poland

## Abstract

The authors discuss the strategy of use of incretin hormones in type 2 diabetes treatment in the context of cardiovascular complications. The results of the phase III study on human GLP-1 (Glucagon-like peptide-1) analogue-liraglutide have been presented under common acronym LEAD (Liraglutide-Effect and Action In Diabetes). The liraglutide therapy improved glycemic control with low hypoglycemia risk and decreased glycated hemoglobin by an average 1,13%. Decreases in systolic pressure and significant body weight loss were observed. Not only did the index describing beta cells function HOMA-B improve but also did the ratio of insulin to proinsulin. Summing up, incretin hormones beneficially influence blood glucose level, moreover, their use decreases blood pressure and body weight which might indicate their positive influence on cardiovascular system in diabetic patients.

## 1. Introduction

Type 2 diabetes is related with the risk of cardiovascular disease development which is the major cause of death [1]. High risk of death and complications increased the interest in introducing therapies which could not only give the possibility of glycemic control but also could influence the cardiovascular parameters. These possibilities give the medications from the group of incretin mimetics. 

The incretin hormones include Glucagon-like peptide-1 (GLP-1) and glucose-dependent insulinotropic peptide (GIP). Intravenous administration of the incretin hormones in patients suffering from type 2 diabetes causes the increase in early-phase insulin secretion in response to a meal, but only GLP-1 is able to stimulate insulin secretion in late phase [2]. It is worth mentioning that the GIP level in patients suffering from type 2 diabetes is similar to the one in healthy people, however, for unknown reasons, it does not influence blood glucose and insulin level. Because of these reasons only GLP-1 has become a basis for new medication development [3]. 

GLP-1 is secreted by L cells in small intestine and duodenum. The secretion of this hormone is stimulated by gastrointestinal passage and digestion process [4]. GLP-1 gets to bloodstream and affects a series of organs via its receptor. From the glycemy regulation point of view, one of the most important elements is the influence on the receptor in beta pancreatic cells. Binding to the receptor stimulates insulin secretion, GLP-1 is responsible for the secretion of 50 to 70% of insulin in response to oral glucose administration [5]. It should be stressed that the insulin secretion stimulation occurs exclusively when glucose level is elevated. Despite the fact that in response to gastrointestinal passage stimulation, quite big amount of GLP-1 hormone is secreted, only a small part of it reach pancreatic islets. This happens because of action of an enzyme dipeptidyl peptidase-4 (DPP-4) which decomposes GLP-1 in blood within 2–4 minutes [6, 7]. 

The strategies of use of incretin hormones in type 2 diabetes treatment include the use of DPP-4 inhibitors and exogenous GLP-1 substitution. Gliptins (DPP-4 inhibitors) are the medications which by DPP-4 enzyme inhibition increase endogenous GLP-1 level. These medications include sitagliptin, wildagliptin and saxagliptin, and other substances undergoing clinical studies at the moment. These medications decrease the level of secreted glucagon and stimulate insulin secretion regulating glycemy [8]. The second therapeutical strategy includes endogenous GLP-1 supplementation, currently registered medications include human GLP-1 analogue (liraglutide, commercial name Victoza) and GLP-1 receptor agonist (exenatide-commercial name Byetta). Exenatide derives from saliva of a lizard Hila Monster, the use of this medication results in body weight loss and decrease in blood glucose level in patients suffering from type 2 diabetes. What is more the use of exenatide resulted in beta cells improvement. The exenatide half-life in human body equals 60–90 minutes and its effective activity is 4–6 hours. Exenatide is administered twice daily [9].

Liraglutide (commercial name Victoza) is an analogue of human GLP-1, in 97% homological with a native molecule. The differences between native GLP-1 and liraglutide include exchange of lysine to arginin in 34 position and addition of glutamate to lysine in position 26; to glutamate also was added 16C fatty acid-palmitic acid [6, 10]. After subcutaneous administration, liraglutide forms heptameres which slow down the release of the substance into the bloodstream. In blood, liraglutide is reversibly connected to albumins by the fatty acids. That is why liraglutide has 24 hour long action. It should be also stressed that introduced modifications protect liraglutide from the action of the DPP-4 [6]. The medication is administered once daily in the doses of 0.6 mg (for at least a week), after a week, the patient should augment the doses to 1.2 mg, which is therapeutic dose, however, it is also possible to take the doses of 1.8 mg.

The III phase of clinical study of liraglutide has common acronym LEAD (Liraglutide Effect and Action in Diabetes) included 4456 people. The LEAD program covered six randomized studies analyzing the influence of drug administration at different therapeutical stages of type 2 diabetes treatment. The summary of the program is presented in Table 1. As it can be noticed, the liraglutide therapy improved glycemic control reducing the level of glycated hemoglobin by an average 1,13%; the reduction depended both on fasting glucose reduction and postprandial glucose reduction [11–16]. During the therapy with this medication, very low risk of hypoglycemia was proved which is related to the mechanism of action of liraglutide, it reduces glycemy only on the condition of elevated blood glucose level [13]. The effect of better glycemic control was accompanied by the reduction in systolic blood pressure at an average by 3,41 mmHg, which can be considered as a protective action from further diabetic complications such as cardiovascular complications [11–16]. The use of liraglutide resulted also in body weight loss and it should be stressed that the observed reduction in arterial blood pressure was independent from the weight loss. Moreover clinical indices describing beta cells function such as HOMA-B and the ratio of proinsulin to insulin were also improved in the whole period of liraglutide usage [14]. This indicates the improvement in beta cells function during liraglutide use. It is also confirmed by preclinical studies which show the liraglutide treatment increase beta cells mass in the animals with artificially destroyed pancreas [17].

The longest study LEAD-3 lasted for 52 weeks, the remaining covered the period of 26 weeks of observation. Part of these studies was prolonged in order to investigate the effects of the long-term effect. The LEAD-6 studies and its prolongation comparing liraglutide to exenatide showed that it is currently the most efficient molecule within the family of GLP-1 receptor agonists [11]. Direct comparison to exenatide in randomized clinical study showed that mean HbA1c change equaled −1,1% in case of liraglutide and −0,8% in case of exenatide whereas the change in FPG equaled 1,61 mmol/L (liraglutide) and 0,6 mmol/L (exenatide). Body weight loss equaled liraglutide—3,24 kg and exenatide—2,87 kg, respectively. It should be stressed that the use of both medications resulted in reduction of systolic blood pressure but, in case of liraglutide, the reduction equaled 2,51 mmHg whereas exenatide reduced systolic blood pressure by 2 mmHg [11]. The prolongation of this study by 14 weeks covered the transition of patients using exenatide to liraglutide therapy. The results of this study showed that the change of medication caused further improvement of glycemic control: the HbA1c reduction, FPG, and, moreover, further body weight loss and systolic blood pressure reduction [18]. 

It should also be stressed that direct comparison of liraglutide with sitagliptin showed advantage of GLP-1 analogue over DPP-4 inhibitor. The changes in HbA1c equaled −1,5% and −1,24% for the doses of 1,8 mg and 1,2 mg of liraglutide, respectively, whereas sitagliptin caused the reduction of HbA1c by −0,6%. During using liraglutide, the body weight loss of −3.38 kg (1,8 mg doses), −2,86 (1,2 mg doses) was observed, the reduction observed during usage of sitagliptin 0,96 kg [19]. The original study lasted 26 weeks and was extended to 1 year. The results of extended study showed further advantage of liraglutide in glycemic control, HbA1c reduction: −1.29% (1,2 mg of liraglutide), −1.51% (1,8 mg of liraglutide) and −0.88% (sitagliptin). Also further differences in body weight loss were observed: liraglutide 1.2 mg (−2.78 kg), 1.8 mg (−3.68 kg), and sitagliptin (−1.16 kg) (*P* < 0.0001). Moreover, patients' treatment satisfaction was higher, when liraglutide in the doses of 1,8 mg was used than sitagliptin [20]. 

The summary of the LEAD program showed that liraglutide therapy cause body weight decreases. The reduction in body weight depends on initial patients' BMI, so body weight reduction might be observed in obese patients, this effect, however, is not observed in patients with normal body weight [15]. Also, preclinical studies performed on rats showed that peripheral liraglutide delivery results in body weight loss in adult obese rats which was related to the reduction of food supply and energy supply [21]. Liraglutide is an analogue molecule to human native GLP-1 and the mechanisms responsible for body weight loss are similar in case of both molecules. These mechanisms also include the influence on digestive system and central nervous system. Liraglutide causes reduction of the gastrointestinal passage and stomach emptying [22]. This is one of the reasons of nausea in about 14% of patients during the first month of the therapy, but it should be pointed that nausea has transitional character [12]. A relationship between the presence of nausea and body weight loss has not been established; body weight loss occurred both in patients experiencing nausea and in those not experiencing it. The influence on nervous system of GLP-1 and liraglutide includes their influence on hunger/satiety centers in central nervous system [21, 23]. Both mechanisms, prolonged stomach emptying and satiety centre stimulation in brain, result in the reduction of food intake and body weight loss. 

Liraglutide despite its obvious influence on body weight is not registered as an antiobesity medication. However, there are studies in which liraglutide action is compared to the medications already registered as anti-obesity medications. In 20 weeks long study, liraglutide was compared to orlisat; the study showed that liraglutide more efficiently reduced body weight in obese patients than orlistat and additionally reduced blood pressure [24]. 

## 2. Liraglutide Influence on Cardiovascular System in Diabetes

Beneficial effect of incretin medications on cardiovascular system results both from direct and indirect influence on heart and arterial system. The indirect effect is the influence on glycemic control with the lack of the risk of hypoglycemia, arterial blood pressure normalization, and body weight loss. Lately, experimental studies have showed that GLP-1 exerts direct beneficial influence on cardiovascular system. 

Liraglutide therapy lasting 26 weeks in patients suffering from type 2 diabetes led to significant cholesterol concentration reduction, including LDL fraction, triglycerides, and free fatty acids. The effect mentioned above was proved in meta analysis of all 6 LEAD studies [25].

Cummings et al. [26] focus on the fact that GLP-1 analogue administered once daily in patients with impaired glucose tolerance or with diabetes de novo decreases postprandial triglyceride, apolipoproteins: B-48, C III, and also cholesterol and triglyceride remnants which results in significant reduction of cardiovascular complication risk factors. According to Jendle et al. [27] except for body weight loss, liraglutide is responsible for the reduction of fatty tissue in body. In this analysis, a dual energy X-ray absorptiometry (DEXA) was used. Fatty tissue reduction was related to reduced production of aterogenous hormones and cytokines by this organ. The authors at the same time confirmed the decrease in fatty liver in patients which beyond any doubt is related to the improvement of sensitivity of this organ to insulin action. 

Plutzky et al. [25] concentrated on the reduction of independent arterial sclerosis risk factors such as C*-*reactive protein (CRP) and brain natriuretic peptide (BNP). The decrease in their concentration inhibits the inflammatory cytokine activation directly engaged in vascular wall destruction. GLP-1 analogues may influence the early stages of aterogenesis by inhibition of monocyte adhesion to the vascular wall. It was shown on animal model deprived of apolipoprotein E. Subsequent *in vivo* studies confirmed liraglutide influence on selected vascular endothelium destruction markers. The use of liraglutide was related to TNF alfa inhibition and hyperglycemia-dependant gene PAI-1, ICAM-1, VCAM-1 induction in the cultures of human endothelial cells. Molecular mechanism of this process has not been explained, however, Liu et al. [28] revealed that liraglutide may regulate expression of mRNA of NUR77 protein. This signal pathway seems to play a key role in endothelial cells protection in type 2 diabetes and in metabolic syndrome. At the same time, observed increase in the production of nitric oxide (NO) and inhibition of NF-*κ*B partially by AMPK (*AMP activate protein kinase*), which documents anti-inflammatory influence of liraglutide on vascular endothelium cells even in hyperglycemia. The effect mentioned above can explain observed earlier vasodilative properties of the medication and its beneficial influence on cardiovascular system in patients suffering from type 2 diabetes. 

In 2009 Noyan-Ashraf et al. [29] confirmed cardioprotective prosperities of liraglutide in animal model. Smaller cardiomyocyte necrosis area in the infarct zone, was observed in mice treated by liraglutide in comparison to mice receiving placebo. Liraglutide use was related to the improvement in survival rate in comparison to placebo (80% versus 40%), with decreased heart wall destruction risk, with reduction of infarct zone and improvement in cardiac output [29]. Those data were confirmed in pilot study where 10 high-risk patients with acute myocardial infarction and systolic dysfunction of left ventricle were subjected to 72 hour long GLP-1 intravenous administration [30]. The authors obtained total increase in left ventricle function which was related to shorter period of hospitalization in comparison to the patients with similar risk factors treated in a standard way. Sokos et al. [31] infused GLP-1 in patients with III-IV NYHA class up to 5 weeks. This allows obtaining a significant improvement in left ventricle function and greater life satisfaction in comparison to the control group. 

The same group showed beneficial influence of liraglutide on the cardioprotective genes activity and expression (Akt, GSK3beta, PPAR-beta-delta, Nrf-2, and HO-1). Cardioprotective effect of liraglutide in comparison to metformin was showed in induced myocardial infarction in animals with diabetes, when similar metabolic control was achieved. This may be explained by liraglutide action on the increase of cAMP concentration and decrease of caspase 3 activation responsible for the cardiomyocyte apoptosis induction. This process was dependent on the GLP-1 receptor [32]. 

In 1995 it was shown that GLP-1 receptors are active in human heart muscle [33]. The studies of the following years were concentrated on the beneficial influence of GLP-1 on left ventricle systolic function, heart muscle reperfusion in chronic ischemia, and also in acute coronary syndrome [29–31, 34, 35]. 

Liraglutide is an efficient medication in the therapy of patients suffering from type 2 diabetes. Retrospective analyses of cardiovascular incidents during liraglutide therapy showed the safety of this medication [36, 37]. From this point of view, the results of currently conducted study LEADER (Liraglutide Effect and Action in Diabetes: Evaluation of Cardiovascular Outcome Results) will be very interesting, the study in which 9000 of patients will be observed for the period of 5 years. In this study, part of the patients will receive liraglutide and part will be subjected to a standard anti- type 2 diabetes therapy. The main aim of this study is the determination of the liraglutide influence on cardiovascular system. When we will have to wait for the outcomes of this study, it is worth to stress that the data cited above suggest cardioprotective action of the incretin based compunds.

##  Conflict of Interests

Ł. Hak is an employee of Novo Nordisk. I. Kinalska, D. Bednarska-Chabowska, and Joanna Adamiec-Mroczek declare no conflicts of interest.

## Figures and Tables

**Table 1:** The summary of LEAD program. FPG—fasting plasma glucose, HbA1c—glycated hemoglobin, SBP—systolic blood pressure.

	*n*	Mean change in HbA1c from initial values (%)	Mean change in FPG from initial values (mmol/L)	Mean change in body weight from initial values (kg)	Mean change of SBP from initial values (mmHg)
LEAD-3 (1573): Liraglutide in monotherapy versus glimepiride					

Liraglutide 1,2 mg	251	−0,8	−0,84	−2,05	−2,12
Liraglutide 1,8 mg	247	−1,1	−1,42	−2,45	−3,64
Glimepirde 8 mg	248	0,5	−0,29	+1,12	−0,69

LEAD-2 (1572): Liraglutide with metformin versus glimepiride					

Liraglutide 1,2 mg	241	−1	−1,64	−2,6	−2,8
Liraglutide 1,8 mg	242	−1	−1,69	−2,8	−2,3
Glimepiride 4 mg	244	−1	−1,31	+1	+0,4

LEAD-1 (1436): Liraglutide with glimeperide versus rosiglitazone					

Liraglutide 1,2 mg	228	−1,1	−1,57	+0,3	−2,6
Liraglutide 1,8 mg	234	−1,1	−1,59	−0,2	−2,8
Rosiglitazone 4 mg	232	−0,4	−0,88	+2,1	−0,9
Placebo	114	+0,2	+1,01	−0,1	−2,3

LEAD-4 (1574): Liraglutide with metformin and rosiglitazone versus placebo					

Liraglutide 1,2 mg	178	−1,5	−2,2	−1	−6,7
Liraglutide 1,8 mg	178	−1,5	−2,4	−2	−5,6
Placebo	177	−0,5	−0,43	+0,6	−1,1

LEAD-6 (1797): Liraglutide with metformin and/or glimepiride versus exenatide					

Liraglutide 1,8 mg	233	−1,1	−1,61	−3,24	−2,51
Exenatide 10 *μ*g	231	−0,8	−0,6	−2,87	−2
